# Melatonin interaction with abscisic acid in the regulation of abiotic stress in Solanaceae family plants

**DOI:** 10.3389/fpls.2023.1271137

**Published:** 2023-09-11

**Authors:** Muhammad Ali, Yupeng Pan, Hanqiang Liu, Zhihui Cheng

**Affiliations:** Department of Vegetable Science, College of Horticulture, Northwest A&F University, Yangling, China

**Keywords:** melatonin, abscisic acid, Solanaceae plants, abiotic stress responses, molecular mechanisms, hormonal interplay, crop improvement, horticultural practices

## Abstract

Solanaceous vegetable crops are cultivated and consumed worldwide. However, they often confront diverse abiotic stresses that significantly impair their growth, yield, and overall quality. This review delves into melatonin and abscisic acid (ABA) biosynthesis and their roles in abiotic stress responses. It closely examines the intricate interplay between melatonin and ABA in managing stress within plants, revealing both collaborative and antagonistic effects and elucidating the underlying molecular mechanisms. Melatonin and ABA mutually influence each other’s synthesis, metabolism and that of other plant hormones, a key focus of this study. The study highlights melatonin’s role in aiding stress management through ABA-dependent pathways and key genes in the melatonin-ABA interaction. Specifically, melatonin downregulates ABA synthesis genes and upregulates catabolism genes, leading to reduced ABA levels. It also directly scavenges H_2_O_2_, enhancing antioxidant enzyme activities, thereby underscoring their collaborative role in mediating stress responses. Moreover, the interplay between melatonin and ABA plays an essential role in multiple physiological processes of plants, including stomatal behaviors, wax accumulation, delay leaf senescence, seed germination, and seedlings growth, among others. Recognizing these relationships in Solanaceae vegetable crops holds great importance for improving agricultural practices and crop quality. In summary, this review offers a comprehensive overview of recent studies on the melatonin and ABA interplay, serving as a valuable resource for researchers and breeders dedicated to fortifying crop resilience and productivity within challenging environments.

## 1 Introduction

### 1.1 Background on abiotic stress in Solanaceae plants

Abiotic stress covers non-living environmental factors that may have varying levels of negative impacts on plants and other organisms. In the present study, the researcher focuses on the negative effects of abiotic stress on a pre-selected plant family, namely, the Solanaceae plant family. Some of the examples of these non-living environmental factors cited in related literature include, but are not limited to, extreme temperatures (hot or cold), flooding, droughts, extreme levels of salinity (or lack thereof), exposure to heavy metals, air pollution, exposure to sunlight, and malnutrition and over-nutrition (Ahammed et al., 2019; Arnao and Hernández-Ruiz, 2019). Abiotic stress is the polar opposite of biotic stress, which is more focused on the stresses caused by and or related to living organisms such as pests, and pathogens (Zhang et al., 2015; Kapoor et al., 2021).

Abiotic stress factors represent a strong challenge to almost all plant families. The only difference, really, is how negatively the plants from these different plant families ought to react to the abiotic stressors present in their environment (Zhang et al., 2022a). In many of the previously published studies about the impacts of abiotic stresses on plant growth and development, however, these negative effects can be attributed to two main factors, namely, their impacts on the target plant and or plant group’s metabolic and physiological processes (Firon et al., 2006; Reddy et al., 2017).

These external stressors are known to cause a cascade of adverse effects on the target plants’ growth, development, and assuming they are being growth for their commercial and other utilitarian purposes, their productivity (Shinozaki and Yamaguchi-Shinozaki, 2007). Some well-known effects of abiotic stress exposure on various plant families, which may well extend to Solanaceae plant family members include, oxidative stress, disruptions in cellular balance and regulation, and impairments in (i.e., suboptimal) nutrient assimilation. All of these lead to alterations in the target plants’ otherwise normal metabolic processes.

### 1.2 Importance of understanding hormonal regulation in stress responses

Hormones, within the context of studying plant physiology and metabolic processes, refer to a group of biochemicals that can significantly impact a target plant’s growth and development (Prasch and Sonnewald, 2013). This impact can either be positive or negative, depending on the exact hormone-related phenomenon. In most if not all cases, any phenomenon categorized as hormonal under- or over-regulation can be expected to have a negative impact on the plant’s growth and development outcomes (Gupta and Huang, 2014). The exact hormone that is causing the problem will also definitely play a role in the exact outcome. For the purposes of establishing the scope and limitations of the present study, the researcher focused on two key plant hormones, namely, melatonin and abscisic acid (ABA). In previously published studies, melatonin and ABA are among the most commonly-studied plant hormones that can impact plants’ responses to abiotic stress factors (Li et al., 2019b; Tiwari et al., 2022). However, they have not particularly been examined with enough focus on the Solanaceae plant family, hence the level of emphasis put on them in this literature review. That said, a comprehension of hormonal regulation within plants forms an essential facet of understanding their responses to abiotic stress.

It is worth noting that plant hormones often work together, forming an intricate network of causes and effects (Arnao and Hernández-Ruiz, 2021; Parwez et al., 2022; Xie et al., 2022a). It is not uncommon for one plant hormone to affect the regulation of other plant hormones, while at the same time, being affected by other plant hormones. This collaborative and sometimes antagonistic interaction further complicate the already complex nature of hormonal regulation. Understanding the dynamics of this interaction, specifically between melatonin and ABA, can unlock new perspectives on how plants respond to and survive under harsh conditions.

The detailed knowledge of hormone regulation can drive the design of innovative plant-breeding strategies, enhancing plant resilience and productivity (Sun et al., 2021; Gu et al., 2022). Furthermore, it can provide insights into precise and efficient application of exogenous hormones in horticultural practices. Unraveling the complexities of hormonal regulation in response to abiotic stress is thus a stepping-stone to more sustainable and productive agricultural systems.

### 1.3 Overview of phytohormones such as melatonin and abscisic acid

Melatonin and ABA form an integral part of the diverse group of plant hormones, each of which play important roles in plant physiology. Originally identified in animals for its function in regulating sleep-wake cycles, melatonin was later discovered to play an important role in plant physiology and metabolism (Banerjee and Roychoudhury, 2019; Hu et al., 2021).

Melatonin serves a collection of functions, including plant growth regulation, reproduction, and regulation of stress responses. Some of its other known functions, according to previously published studies, include but may not be limited to the mitigation of oxidative damage, enhancement of antioxidant defenses, and modulation of several other hormones’ activity, illustrating its pervasive influence (Tan et al., 2019; Hosseini et al., 2021; Singh et al., 2022).

The second phytohormone highlighted in this review is Abscisic Acid, a sesquiterpene synthesized through the carotenoid pathway, and recognized as a stress hormone (Mialoundama et al., 2009; Moreira et al., 2020). This reputation can be attributed to the integral role it plays in regulating plant responses to stressful conditions, primarily drought and salinity. It regulates critical processes such as stomatal closure to reduce water loss and promotes the synthesis of protective proteins. Moreover, ABA serves crucial roles in seed dormancy and germination, demonstrating its diverse functions.

Despite the notable differences in their functions, these hormones cannot be considered solitary actors. They function within a complex network of phytohormone functions, influencing, and being influenced by, the activity of other hormones. The interplay between melatonin and ABA under abiotic stress conditions is a compelling aspect of this network, warranting detailed exploration for its potential implications in horticulture (Mukherjee, 2018; Lv et al., 2021; Altaf et al., 2023b; Singh and Roychoudhury, 2023).

### 1.4 Significance of studying the interactions between phytohormones such as melatonin and abscisic acid in Solanaceae plants

Exploring the intricate interactions between melatonin and ABA in Solanaceae plants holds substantial promise for improving our current level of understanding of abiotic stress response mechanisms in plants. The Solanaceae family, encompassing economically and nutritionally significant species such as tomatoes, potatoes, and peppers, is particularly susceptible to substantial productivity losses caused by environmental stresses (Beilby et al., 2015; Murch and Erland, 2021; Altaf et al., 2022a; Gao et al., 2023). This clearly illustrates the importance of furthering our understanding of the molecular and physiological mechanisms behind stress response in plants.

The interplay of melatonin and ABA under stress conditions is not merely a dyadic relationship but forms a part of a comprehensive and complex network. Their interactions might involve synergistic, antagonistic, and complex regulatory pathways, making them an interesting point of inquiry in this field. An improved understanding in this area could, for example, reveal how the crosstalk between these hormones can be manipulated to enhance plant tolerance to various abiotic stresses.

Moreover, comprehending the roles and interactions of these hormones can potentially lead to the development of new biotechnological strategies for crop improvement. These could encompass the genetic manipulation of key players in the melatonin and ABA pathways or the use of exogenous hormone applications to fortify plants against adverse conditions. Consequently, the implications of this research extend far beyond basic plant biology, promising to unlock novel, efficient strategies for enhancing crop resilience and productivity.

## 2 Melatonin: roles in abiotic stress responses

### 2.1 Overview of melatonin biosynthesis and metabolism

**Figure 1:**
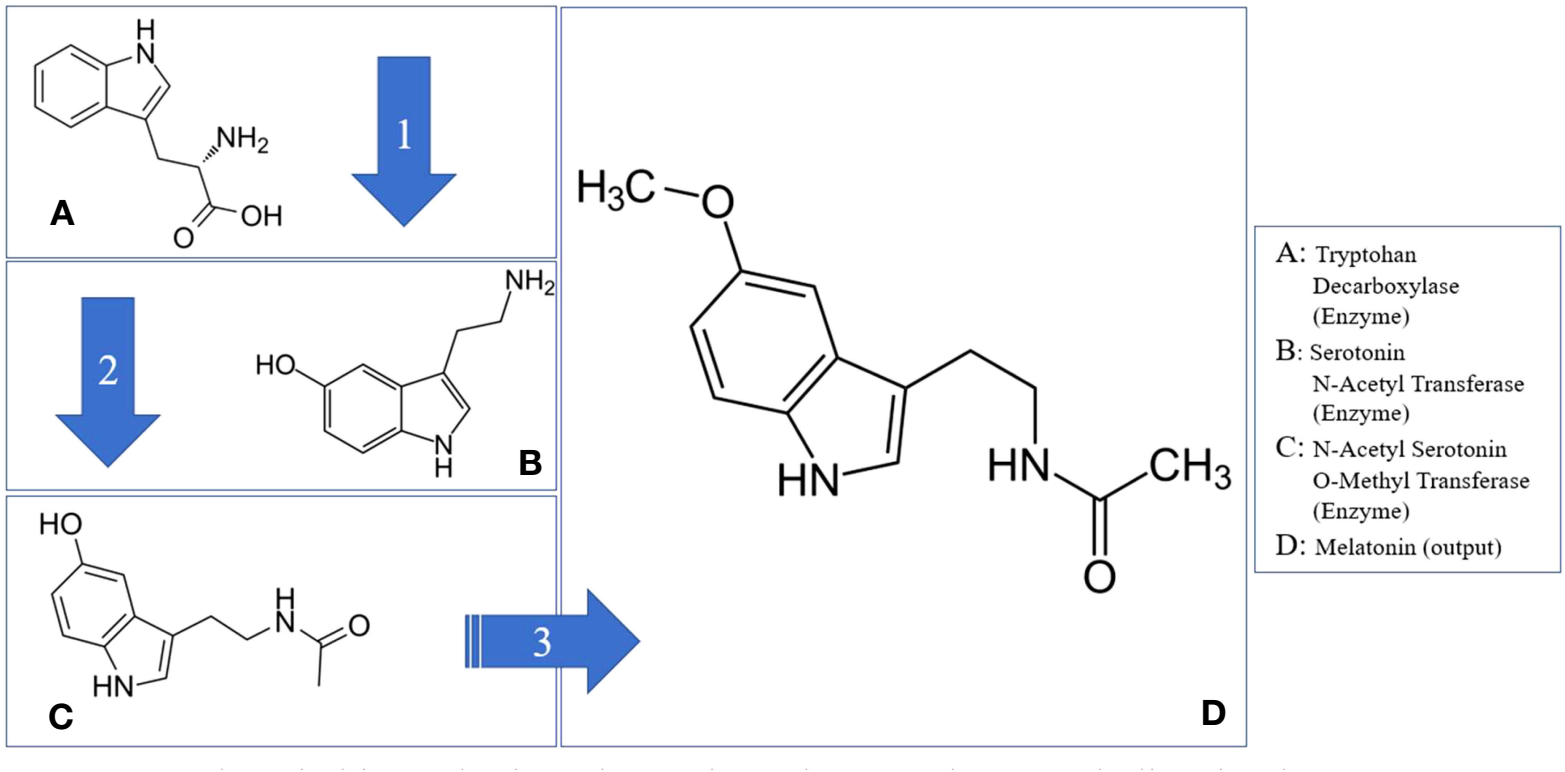
Melatonin biosynthesis pathway through tryptophan metabolism in plants. Tryptophan Decarboxylase (Enzyme); N-Acetyl Transferase (Enzyme); N-Acetyl Serotonin O-Methyl Transferase (Enzyme); Tryptophan (Hormone Precursor); Serotonin (Hormone); Melatonin (Hormone); N-Acetyl Serotonin (Hormone Precursor).

Melatonin synthesis in plants involves a series of enzymatic reactions, which may vary in the number of steps according to different sources. To ensure comprehensiveness, these reactions are categorized into four phases (**Figure 1**). The primary enzyme driving this process is Serotonin N-Acetyltransferase (SNAT) (Back et al., 2016; Zhao et al., 2019; Tan and Reiter, 2020).

However, it is important to note that SNAT isn’t the starting point of melatonin biosynthesis in plants; that role belongs to tryptophan. Tryptophan is an amino acid, acts as a precursor to melatonin. The plant secretes the enzyme tryptophan decarboxylase (TDC), which triggers a chemical reaction with tryptophan, converting it into serotonin. This isn’t the final step in melatonin synthesis. The subsequent phase involves converting serotonin into N-Acetyl Serotonin (NAS), achieved when the enzyme serotonin N-Acetyl Transferase (SNAT) interacts with serotonin (Posmyk et al., 2009; Wang et al., 2017). The final phase of the melatonin biosynthesis involves converting NAS into melatonin, initiated by the enzyme N-Acetyl Serotonin O-Methyl Transferase (ASMT) (Byeon et al., 2015; Wei et al., 2017; Zhou et al., 2021).

The metabolic phase of melatonin in plants differs significantly in how synthesized melatonin is utilized and consumed. Additionally, there are multiple pathways for melatonin metabolism, generally categorized into three variations.

The first pathway involves converting synthesized melatonin into 2-Hydroxymelatonin, requiring the presence of the enzyme melatonin 2-hydroxylase. The second pathway breaks down melatonin into N-acetyl-5-methoxykunuramine (AMK) which involves enzymes like cytochrome P450. The third pathway involves melatonin’s conversion into N-Acetyl-Serotonin, or other metabolites, such as N-acetyl-3-hydroxykunuramine (Arnao et al., 2021; Back, 2021).

An important aspect that industry practitioners and researchers should consider regarding melatonin biosynthesis and metabolism is the factors influencing these processes. In general, melatonin biosynthesis and metabolism can be influenced by exposure to factors like environmental stresses, temperature, and even light, i.e., abiotic stressors (Hardeland, 2016; Jahan et al., 2021a; Mannino et al., 2021; Liu et al., 2022). For instance, a plant’s melatonin levels fluctuate throughout the day due its exposure to light, with the highest levels of melatonin observed in the plant system occurring during night time.

It’s noteworthy that the specific biosynthetic and metabolic processes, including pathways, can vary based on the plant family or species (Sharma et al., 2020; Xie et al., 2022b). This emphasizes the important of focusing on a specific plant family during studies to avoid confusing mechanisms and reactions related to melatonin synthesis and metabolism.

Melatonin plays a pivotal role in regulating plant physiological processes linked to stress response. Its primary function, widely recognized, is acting as an antioxidant (Khan et al., 2020; Pardo-Hernández et al., 2020). While various antioxidants exit, that plants rely on to alleviate abiotic stress and oxidative damage, such as polyphenols and vitamins (Gu et al., 2022). Melatonin stands out due to its potent efficacy in minimizing oxidative harm to the plant system than its counterparts (Altaf et al., 2023a). In addition to its antioxidant function, melatonin also impacts the regulation of stomatal opening. Under stress conditions like drought, melatonin-induced stomatal closure helps reduce water loss, conserving moisture and contributing to drought resistance (Ahmad et al., 2021; Rajora et al., 2022). Likewise, melatonin makes a substantial contribution to the mitigating of salt stress by balancing of the Na^+^/K^+^ homeostasis inhibition through the coordination action of carbohydrate and nitrogen metabolism in tomato seedlings (Jahan et al., 2023).

Melatonin significantly influences the plant system’s expression of stress-responsive genes (Ali et al., 2023; Colombage et al., 2023). These genes often directly involved in the plant system’s defense mechanisms. Additionally, melatonin plays a crucial role in modulating signals transmitted by other plant hormones that manage abiotic stress responses. This highlights melatonin’s importance in hormonal crosstalk, or the phenomenon characterized by the interconnection of the activities of various hormones in a plant system, be it synergistic, or antagonistic, or a combination of both, among others.

Furthermore, melatonin aids in maintaining energy homeostasis under stress conditions (Moustafa-Farag et al., 2020; Zhang et al., 2022b). It optimizes photosynthetic efficiency and adjusts the energy metabolism pathways, facilitating energy conservation and survival under adverse conditions. Thus, melatonin’s multifaceted involvement in stress-related physiological processes highlights its potential for enhancing plant resilience against various abiotic stresses.

### 2.2 Melatonin’s impact on Solanaceae plants under various abiotic stressors

Melatonin plays a pivotal role in Solanaceae plants, including species such as tomatoes, potatoes, and eggplants, actively mitigating a variety of abiotic stresses (Debnath et al., 2018; Yu et al., 2018; Song et al., 2023; Zhou et al., 2023). Research has demonstrated its ability to fortify these plants against various adverse environmental conditions. For instance, during periods of drought stress, melatonin has been shown to stimulate stomatal closure in tomato plants, thereby reducing water loss and increasing their drought tolerance (Jensen et al., 2023). In conditions of excessive salinity, melatonin assists in maintaining the ion balance, preventing harmful accumulation of sodium ions in tomato and pepper plants (Li et al., 2019b; Altaf et al., 2021).

Additionally, melatonin has been identified as an effective agent against cold stress in potato plants (El-Yazied et al., 2022; Efimova et al., 2023). Its application has resulted in an increase in the levels of antioxidants, helping the plants to manage oxidative damage that often occurs under cold conditions (Korkmaz et al., 2021). In the face of heavy metal toxicity, an increasing concern in agricultural practices, melatonin aids in chelating these metals, thereby reducing their harmful effects on Solanaceae plants (Jahan et al., 2020; Altaf et al., 2022b; Altaf et al., 2023a). These examples underscore melatonin’s protective function in Solanaceae plants under diverse abiotic stressors. This knowledge can potentially be harnessed to improve the resilience and yield of these economically important crops in stressful environments.

## 3 Abscisic acid: roles in abiotic stress responses

### 3.1 Overview of ABA biosynthesis and signaling pathway

**Figure 2:**
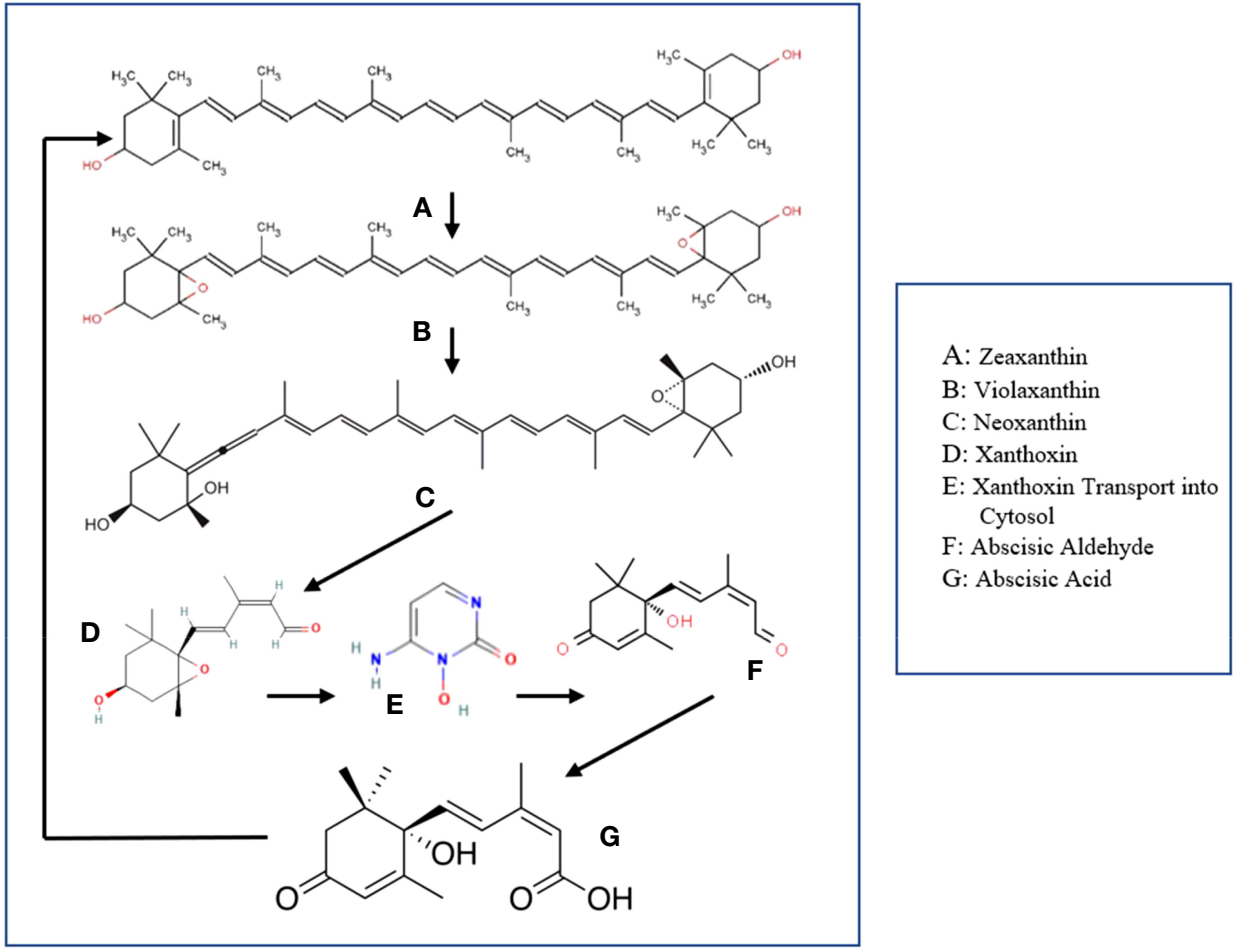
ABA biosynthesis and signaling pathway. Zeaxanthin – ABA Precursor; Violaxanthin - Intermediate output in ABA biosynthesis; Neoxanthin - Intermediate output in ABA biosynthesis; Xanthoxin - Intermediate output in ABA biosynthesis; Abscisic Aldehyde – Intermediate output in ABA biosynthesis; Abscisic Acid - Output.

Abscisic Acid is a principal phytohormone widely recognized for its potency to directly influence plant physiological processes, even at minimal concentrations (Chen et al., 2020). ABA synthesis begins within plant plastids using zeaxanthin as a precursor (**Figure 2**). Zeaxanthin reacts with zeaxanthin epoxidase, producing violaxanthin, then neoxanthin (Hauser et al., 2011). The next part of the process involves the cleaving of the formed neoxanthin into xanthoxin, via the enzyme epoxy carotenoid dioxygenase (NCED). Xanthoxin enters the cytosol, converting to abscisic aldehyde through xanthoxin dehydrogenase. Finally, the resulting abscisic aldehyde gets converted into what we now know as the Abscisic Acid, via the enzyme abscisic aldehyde oxidase (Xiong and Zhu, 2003; Cardoso et al., 2020).

Once synthesized, ABA can be perceived by a group of proteins known as PYR/PYL/RCAR receptors in the presence of negative regulators known as protein phosphatase 2Cs (PP2Cs). Formation of the ABA-PYR/PYL/RCAR-PP2C complex inhibits PP2C activity, activating SnRK2s protein kinases. Once activated, SnRK2s can phosphorylate various downstream targets including ion channels and transcription factors, thus translating the ABA signal into appropriate physiological responses.

This ABA biosynthesis and signaling pathway significantly influences plant responses to abiotic stresses, such as drought, salinity, and cold, by regulating processes like stomatal closure, seed dormancy, and induction of stress-responsive genes (Tuteja, 2007; Liu and Hou, 2018). Thus, comprehending this pathway offers valuable insights into abiotic stress tolerance mechanisms in plants.

### 3.2 ABA-mediated regulation of stress-related physiological processes

Abscisic Acid is pivotal in the orchestration of plant responses to a variety of abiotic stresses. It influences several stress-related physiological processes which allow the plant to adapt and survive in challenging environments. Notably, ABA governs stomatal movement, a crucial mechanism. Under conditions such as drought or high salinity, ABA triggers stomatal closure, reducing water loss via transpiration and thereby improving plant water use efficiency (Romero-Romero et al., 2005; Easlon and Richards, 2009; McAdam et al., 2017).

Furthermore, ABA mediates adaptive cellular responses, particularly in maintaining ion balance during salt stress. By regulating transporters and channels, ABA helps to maintain cellular ion homeostasis, preventing the toxic accumulation of sodium ions in cells. Another vital role of ABA is the induction of stress-responsive genes. It activates a wide array of genes encoding proteins that bolster the antioxidant system, assist in osmotic adjustment, and synthesize heat-shock proteins for protection under heat stress (Palchetti et al., 2021; Saidi et al., 2021). Lastly, ABA is involved in the regulation of seed dormancy and germination, allowing plants to synchronize their life cycle with environmental conditions. It prevents premature germination under adverse conditions, thereby increasing the chances of seedling survival. These varied regulatory roles underscore the importance of ABA in modulating physiological responses to abiotic stresses in plants.

### 3.3 ABA’s impact on Solanaceae plants under different abiotic stress conditions

Abscisic Acid has a remarkable impact on Solanaceae plants, enabling them to combat various abiotic stresses. Through its regulatory functions, ABA shapes adaptive responses in these plants amidst challenging environments (León et al., 2001; Nivedithadevi et al., 2012). For instance, during drought stress, ABA promotes stomatal closure in tomato plants, thereby conserving water and enhancing their drought tolerance. Simultaneously, it triggers the expression of drought-responsive genes, further equipping the plant to withstand water scarcity.

In response to salt stress, ABA helps modulate ion homeostasis in Solanaceae plants like pepper, tomato and eggplant. It orchestrates the regulation of ion channels and transporters to prevent toxic accumulation of sodium ions and maintain cellular stability under high salinity (DiLeo et al., 2010; Ruggiero et al., 2019; Meng et al., 2020; Alkhatib et al., 2021).

Cold stress is another challenge where ABA has shown its influence. In potato and pepper plants, an increase in ABA levels under cold conditions has been linked to enhanced cold tolerance, likely due to the activation of cold-responsive genes (Guo et al., 2013; Danieli et al., 2023).

Moreover, ABA plays a crucial role in managing oxidative stress in Solanaceae plants. Its involvement in strengthening the antioxidant defense system helps mitigate the harmful effects of reactive oxygen species (ROS) generated under various abiotic stress conditions. These adaptive responses, modulated by ABA, underscore its integral role in abiotic stress resilience in Solanaceae plants, opening avenues for leveraging this hormone in improving crop performance under stressful environments (Back, 2021).

## 4 Interactions between melatonin and ABA in abiotic stress responses

### 4.1 Crosstalk between melatonin and ABA signaling pathways

The interplay between melatonin and ABA is pivotal for a plant’s regulatory processes during abiotic stress. Their intersecting signaling pathways form a sophisticated regulatory network that aids plant survival under challenging conditions (Hu et al., 2021). Notably, they collaboratively influence stomatal responses, with melatonin enhancing ABA’s role in promoting stomatal closure to counter water-deficit stress (Li et al., 2015; Prakash et al., 2019).

On a molecular level, melatonin impacts ABA-responsive gene expression, potentially fine-tuning stress responses (Asad et al., 2019; Zhang et al., 2023). Furthermore, there is evidence that ABA itself can impact melatonin production in plants. Some studies suggest an uptick in melatonin synthesis following ABA application, indicating an interactive feedback loop between these signaling molecules (Zhang et al., 2014).

These insights underline the complex dialogue between melatonin and ABA in the signaling network of plants. Gaining a deeper understanding of these interactions may offer innovative perspectives on hormonal control of abiotic stress responses in plants.

### 4.2 Cooperative effects of melatonin and ABA in stress tolerance

The collaboration between melatonin and ABA plays a significant role in augmenting plant tolerance to abiotic stresses. The cooperative action of these two compounds orchestrates multifaceted adaptive responses.

Drought stress provides a prime example of this cooperation. Both melatonin and ABA contribute to regulating stomatal closure, an essential adaptive mechanism to conserve water. Studies suggest that melatonin can bolster ABA-induced stomatal closure, creating a more effective response to water scarcity (Li et al., 2015; Yoon et al., 2019; Dai et al., 2020).

In the face of high salinity, the duo demonstrates another cooperative action. Melatonin has been found to boost the ABA-mediated regulation of ion channels and transporters (Zhang et al., 2014). This synergy enhances the plant’s ability to maintain ion balance, enabling survival in salt-stressed environments. Moreover, melatonin treatments elevate ABA, H_2_O_2_, and ethylene, facilitating berry ripening (Xu et al., 2018). Furthermore, both compounds work together in regulating gene expression under stress conditions. Melatonin affects the expression of ABA-responsive genes, suggesting a cooperative role in gene regulation that enhances the plant’s stress tolerance.

These cooperative effects between melatonin and ABA provide an exciting avenue for further research. A deeper understanding of their interactions could provide new strategies for improving crop resilience in challenging environmental conditions.

### 4.3 Antagonistic effects and regulatory balance between melatonin and ABA

Melatonin and ABA often work together, they also showcase contrasting behaviors in certain situations, emphasizing a delicate balance in plant stress responses. A key area where this balance comes into play involves plant growth and development (Arnao and Hernández-Ruiz, 2018; Arnao et al., 2021). While ABA generally suppresses growth under stress conditions, melatonin can counteract this by promoting growth and development. This interplay between melatonin and ABA helps to maintain a balance between growth and stress defense.

Another antagonistic interaction is observed in controlling plant transpiration. Whereas ABA promotes stomatal closure to reduce water loss under drought conditions, melatonin can counter this action by stimulating stomatal opening under certain light conditions, regulating the plant’s water usage and photosynthetic activity (Yan et al., 2020). At the molecular level, melatonin selectively down-regulates the ABA synthesis gene *MdNCED3*, while up-regulating the catabolic genes *MdCYP707A1* and *MdCYP707A2*, resulting in reduced ABA levels during drought stress. Additionally, melatonin acts as an antioxidant by directly scavenging H_2_O_2_ and enhancing the activity of antioxidant enzymes for H_2_O_2_ detoxification (Li et al., 2015). Under salt stress, melatonin upregulates the expression of ABA catabolism genes (*CYP707A1* and *CYP707A2*), while downregulated the key enzymes (NCED) involved in ABA biosynthesis, thereby decreasing ABA content (Zhang et al., 2014). During heat-stress, the application of exogenous melatonin delays the senescence process by reducing ABA content in plants (Jahan et al., 2021b). Furthermore, melatonin promotes seed germination by antagonizing ABA and regulating the balance between ABA and gibberellic acid (Li et al., 2021).

This regulatory balance between melatonin and ABA, featuring both cooperative and antagonistic interactions, underscores the complexity of hormonal control in plant stress responses, providing rich avenues for further exploration.

### 4.4 Molecular mechanisms underlying the melatonin-ABA interaction in stress responses

Delving deeper into the biological processes at the heart of plant stress responses, we find complex molecular mechanisms orchestrating the interaction between melatonin and ABA. These mechanisms govern the interplay between these hormones and ultimately dictate plant responses to a wide array of abiotic stressors. A prominent facet of this interaction lies in the modulation of gene expression (Zhang et al., 2023). It is well-documented that both melatonin and ABA can influence the transcription of numerous stress-related genes, adjusting plant responses accordingly. Notably, melatonin has been shown to augment the expression of several ABA-responsive genes, implying a degree of synergy at the transcriptional level (Tan et al., 2019). This might involve both hormones binding to their respective receptors and activating downstream transcription factors, which then collectively alter gene expression to facilitate adaptive responses to stress.

The interaction between melatonin and ABA also extends to the realm of signal transduction. Studies suggest that melatonin can modulate various components of the ABA signaling pathway (Hardeland, 2015). Specifically, melatonin has been linked to the regulation of protein kinases and phosphatases within the ABA signaling cascade. This could imply that melatonin doesn’t merely operate in parallel to ABA, but intricately intertwines with the ABA signaling network at several junctures, effectively influencing its output. Interestingly, recent research points towards the possibility of even more direct interactions between melatonin and ABA at the cellular level (El-Yazied et al., 2022). Certain studies propose that melatonin might interact with ABA or its metabolites within plant cells. Though this premise still requires further investigation, it heralds intriguing avenues for future exploration. An excellent example of a plant gene affected by both melatonin and ABA, specific to the Solanaceae plant family, is *SIDREB2*. Melatonin modulates the expression of this gene, while ABA stimulates its expression. *SIDREB2* is a variant of *DREB2*, a dehydration-repressive element-binding Protein responsible for regulating the plant’s response during drought conditions (Shi and Qian, 2015). Studying this antagonistic response between melatonin and ABA in tomatoes, it was found that during drought conditions, exogeneous hormones such as ABA stimulate gene expression, while melatonin works by modulating it (Hichri et al., 2016).

Another example emerges within the study, where complex signals govern the pivotal stage of seed germination in the plant lifecycle. Exogenous melatonin has been demonstrated to influence seed germination, especially in stressful conditions. Field experiments have shown that melatonin priming enhances morphological traits, seed quality, and yield in water-deficient arid cropping systems (Wang et al., 2020). When applied to cucumber (*Cucumis sativus* L.) seeds during chilling stress, melatonin in the range of 25–100 μM boost germination rates, seedling growth, and crop production (Posmyk et al., 2009). In situations involving ABA stress, melatonin fosters the germination rate of melon seeds (*Cucumis melo* L.) by increasing GA_3_ content and decreasing ABA content (Li et al., 2021). However, in contrast, (Lv et al., 2021) found that melatonin concentrations of 10 or 100 μM do not impact seed germination, while 500 or 1000 μM significantly hinder germination by elevating ABA and indole-3-acetic acid (IAA) levels under normal conditions. Notably, melatonin exhibits divergent effects on seed germination in stressful and normal conditions, possibly due to interactions with other hormones like ABA, GA, and auxin in varying environmental contexts. Future research studies should explore these interactions between melatonin and plant hormones during seed germination following melatonin treatment.

Furthermore, post-translational modifications (PTMs) such as phosphorylation, ubiquitination, and sumoylation play a vital role in the interaction between melatonin and ABA (Zhou et al., 2011). They modulate the stability, subcellular localization, and activity of signaling proteins in the melatonin and ABA pathways, fine-tuning the stress response. Additionally, the roles of secondary messengers, such as calcium ions and reactive oxygen species, in the melatonin-ABA interaction deserve attention. These molecules participate in signal amplification and regulation of several physiological processes related to stress responses.

### 4.5 Molecular interaction between melatonin and ABA in ROS homeostasis

Melatonin and ABA interact in intricate ways to mediate plant responses to various stresses. This interplay can significantly influence ROS homeostasis, crucial for mitigating the oxidative damage caused by environmental stressors (Arnao and Hernández-Ruiz, 2019; Hu et al., 2021). Under stress conditions, ABA acts as a central stress hormone that triggers plant responses, including stomatal closure and the expression of stress-response genes. This often leads to an increase in ROS production, contributing to oxidative stress. However, excessive ROS can be harmful and needs to be efficiently scavenged (Cutler et al., 2010; Guo et al., 2023).

This is where melatonin plays a vital role. Recognized for its powerful antioxidant properties, melatonin directly scavenges ROS and also stimulates the activities and gene expression of antioxidant enzymes, thus contributing to ROS homeostasis (Arnao and Hernández-Ruiz, 2021). It triggers genes such as respiratory burst oxidase (*RBOH*), heat shock transcription factors A2 (*HsfA_2_*), heat shock protein _90_ (*HSP_90_*), and delta _1_-pyrroline-_5_-carboxylate synthetase (*P_5_CS*). These genes collectively aided in reducing excessive ROS through the hydrogen peroxide-mediated signaling pathway for detoxification (Jahan et al., 2019). Interestingly, ABA can also stimulate melatonin production in plants, potentially as a means to regulate the ROS increase initiated by ABA itself (Guo et al., 2023).

Further, the interplay between ABA and melatonin impacts the complex signaling networks involved in plant stress responses. The modulation of ROS production and scavenging by these two hormones can influence the MAPK signaling pathway and the expression of stress-responsive genes, thus coordinating plant adaptations to adverse conditions (Raja et al., 2017). The interaction of melatonin and ABA, specifically concerning ROS homeostasis, offers fascinating insights into plant stress biology. It underscores the importance of hormonal interplay and ROS control in shaping plant responses to environmental stresses.

In sum, the molecular interplay between melatonin and ABA under stress conditions is a marvelously intricate affair, with multiple layers of regulation and interaction. From gene transcription and signal transduction to possible direct cellular interactions and the role of PTMs and secondary messengers, there are numerous molecular mechanisms at play. Each adds to our understanding of how these hormones collaborate or compete, shedding light on the enigma of plant stress responses and presenting valuable insights for future research.

## 5 Experimental evidence and findings

### 5.1 Studies investigating the melatonin-ABA interaction in Solanaceae plants and reported effects on plant physiology, biochemistry, and molecular responses

Numerous investigations have explored the intricate relationship between melatonin and ABA in different plant families, contributing unique perspectives to our understanding (**Table 1**).

**Table 1:** Melatonin-ABA cross-talk studies in different crops.

Family	Latin name	Melatonin accumulation	organ	Response observed	Refs.
Rosaceae	*Malus domestica*	10–500 μM, 24 h	leaf	↓ABA ↓ABA biosynthesis genes ↑ABA catabolism genes	(Li et al., 2015)
Rosaceae	*Prunus avium*	10–100 μM, 19 days	fruit	↑Zeatin, ↓anthocyanin level, delay ripening	(Tijero et al., 2019)
Rosaceae	*Malus domestica*	1.3 μM, 1–20 days	seedlings	↑Rooting, ABA level and signaling	(Mao et al., 2020)
Brassicaceae	*Brassica rapa*	100 μM, 1–5 days	leaf	↓Leaf senescence ↓ABA biosynthesis genes	(Tan et al., 2019)
Cucurbitaceae	*Cucumis melo*	10 mM	seed	ABA and GA_3_ balance Melatonin counteracts ABA to induce seed germination	(Li et al., 2021)
Cucurbitaceae	*Cucumis melo*	100 μM,	leaf	↑ *CmRBOHD* under ABA‐induced stress ↑ H_2_O_2_ and ↑ Ca^2+^ signaling delays leaf senescence	(Guo et al., 2023)
Cucurbitaceae	*Citrullus lanatus*	100–150 μM, leaves/roots	fruit	↑ ABA levels, ↑ wax accumulation ↑drought tolerance	(Li et al., 2019a)
Cucurbitaceae	*Cucumis sativus*	10–500 μM, 24 h	seed	↓ABA ↓ABA biosynthesis genes ↑ABA catabolism genes Alleviates salinity stress	(Zhang et al., 2014)
Cucurbitaceae	*Cucumis sativus*	10 μM, 6 h	seed	↓ABA, ↑GA preventing *CsPYL*, ↑activity of *CsPP2C* Under low temperature stress	(Zhang et al., 2023)
Vitaceae	*Vitis vinifera*	10–100 µM	fruit	↑ABA, ↑H_2_O_2_, ↑ethylene production, promoted berry ripening	(Xu et al., 2018)
Juglandaceae	*Carya cathayensis*	100 μM, 5 days	fruit	Drought tolerance, ↓ ABA level	(Sharma et al., 2020)
Anacardiaceae	*Mangifera indica*	0.5 mM, 1 h	fruit	↓ABA level, NCED, ethylene, ripening	(Liu et al., 2020)
Solanaceae	*Solanum tuberosum*	0.1 mM	tuber	↓ABA, ↓Proline, ↑SOD, ↑CAT, ↑APX, ↑GPX	(El-Yazied et al., 2022)
Solanaceae	*Solanum* *lycopersicum*	50 μM	seedlings	↑antioxidant capacity, ↓ROS, ↓MDA ↓ABA content ↓ABA biosynthesis genes ↑ABA catabolism genes ↑AsA-GSH cycle Salt stress tolerance	(Hu et al., 2021)
Solanaceae	***Solanum*** *lycopersicum*	foliar spray 100 µM, every 2 days cont. for 7 days	seedlings	↑Fv/Fm ratio, ↓ROS production ↓*Rbohs* gene, ↓chlorophyll catabolic genes, ↓senescence-associated gene, ↑GA content, ↓ABA content Heat tolerance	(Jahan et al., 2021b)

Melatonin promotes cucumber seed germination under high salinity by regulating antioxidant systems, and ABA-GA_4_ interaction (Zhang et al., 2014). Melatonin upregulates ABA catabolism genes (*CsCYP707A1* and *CsCYP707A2*) and downregulates ABA biosynthesis genes (*CsNECD2*), leading to a rapid decrease in ABA content during early germination. This discovery suggests melatonin’s potential in enhancing plant resilience to salinity by balancing internal hormones and antioxidant enzyme activities. However, it’s essential to note that this study focused on cumber, a plant from a different family.

In a study by (Zhang et al., 2023), the regulation of ABA biosynthesis and catabolism by melatonin during seed germination under low-temperature stress in cucumber was investigated. Although cucumber is not a Solanaceae plant, this study provided valuable insights into the potential molecular mechanisms of melatonin and ABA interaction. The researchers proposed that melatonin reduces ABA content, preventing CsPYL (*CsPYL1/2/3/8/10*) from binding to CsPP2C thus enhancing the activity of CsPP2C (*CsPP2C3/5*) and blocking *CsSnRK2.1* activation, which ultimately leads to seed germination under low temperature stress.

In another significant research effort (Tan et al., 2019), suggested that exogenous melatonin treatment in Chinese flowering cabbage prolonged storage-induced leaf senescence by inhibiting ABA production and reducing Chl reduction, associated with ABA signaling transcription factors, namely *BrABF1*, *BrABF4*, and *BrABI5*. Although Chinese flowering cabbage is not a Solanaceae plant, this provides additional insights into the interactive roles of melatonin and ABA in plant stress responses.

In another pivotal work, (Guo et al., 2023) highlighted melatonin’s ability to counteract ABA’s effects and delay leaf senescence. Through their study on melon plants, they found that melatonin induced the accumulation of H_2_O_2_ and upregulated the expression of *CmRBOHD* under ABA-induced stress. Both melatonin and H_2_O_2_ triggered cytoplasmic-free Ca^2+^ increase in response to ABA. Blocking Ca^2+^ influx channels attenuated melatonin and H_2_O_2_-induced ABA tolerance, indicating a collaborative relationship between these hormones in mediating stress responses.

**Figure 3:**
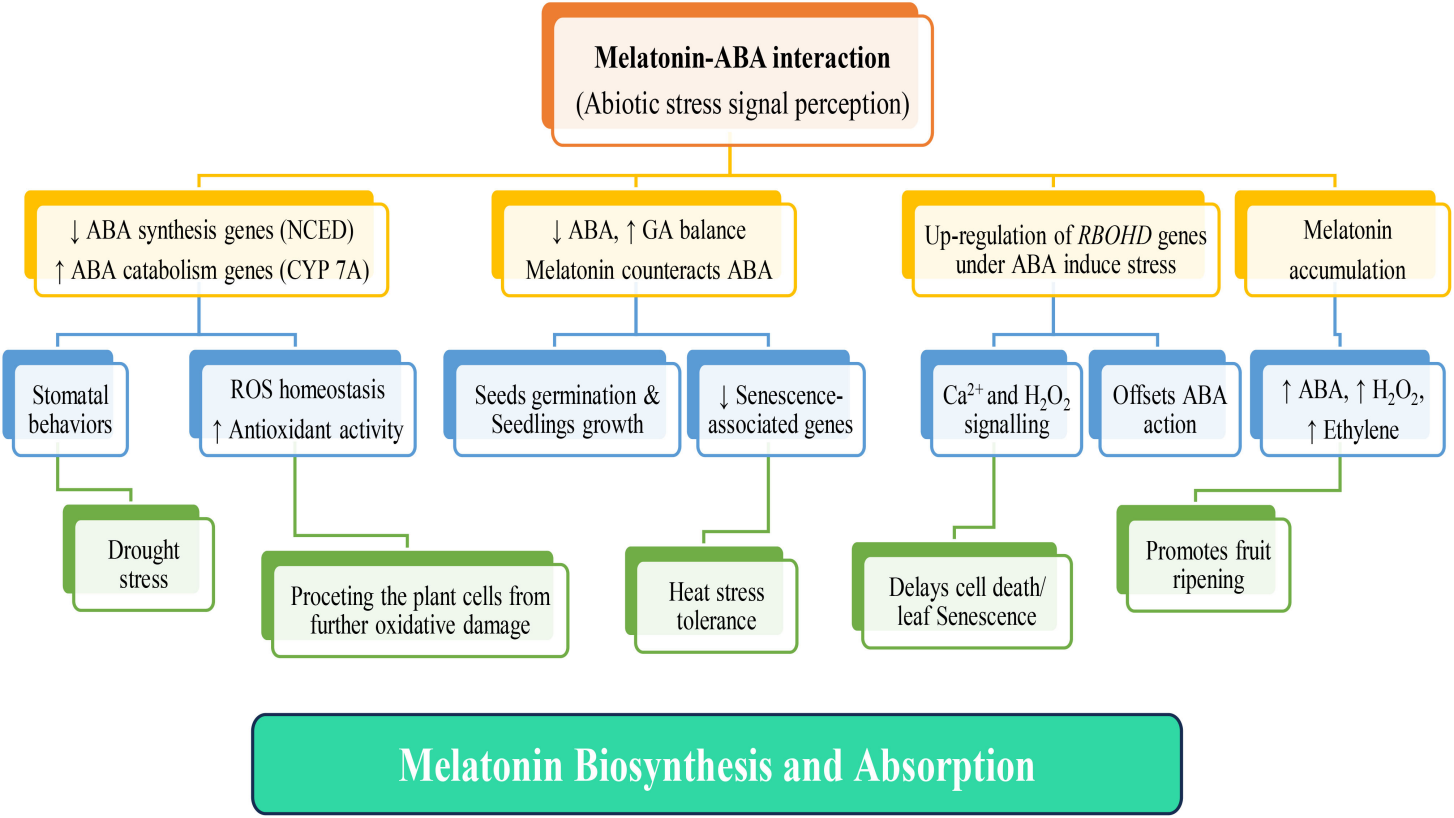
Interplay of melatonin and ABA in the plant systems under various abiotic stress signal perceptions. Upon exposure of plants to different abiotic stresses, their receptors initiate stress signal transduction, triggering subsequent melatonin production and absorption. Elevated melatonin levels enhance antioxidant activity, facilitating the scavenging of reactive oxygen species (ROS). This defensive mechanism shields plants systems against oxidative damage, and retarding leaf senescence. The response varies based on the specific abiotic stressor; stress-related gene modulation occurs, involving genes such as ABA synthesis-related *NCED*, ABA catabolism-associated *CYP7A*, and *RBOHD* genes, among others. Melatonin plays a pivotal role in phytohormone regulation, influencing the secretion of various plant hormones, including ABA (Abscisic Acid), Ethylene, and GA (Gibberellin). This multifaceted hormone orchestrates processes such as stomatal closure in response to drought, enhancement of heat stress tolerance, promoting seed germination and seedling growth by balancing ABA and GA, and promotion of fruit ripening via upregulating of ABA, H_2_O_2,_ and Ethylene signaling pathways.

Lastly, in a study by (Li et al., 2015) investigated how melatonin influences ABA metabolism, free-radical scavenging, and stomatal behaviors in two *Malus* species under drought stress. They found that melatonin down-regulated *MdNCED3*, an ABA synthesis gene, while up-regulated catabolic genes, *MdCYP707A1* and *MdCYP707A2*, resulting in reduced ABA contents in drought-stressed plants. Additionally, melatonin directly scavenged H_2_O_2_ and enhanced antioxidant enzyme activities, indirectly detoxifying H_2_O_2_. The study also revealed that plants effectively regulated their water balance under drought conditions by up-regulating the expression of melatonin synthesis genes *MdTDC1*, *MdAANAT2*, *MdT5H4*, and *MdASMT1*. This response was mediated through melatonin-ABA cross-talk, further reinforcing the idea of these two molecules functioning together to address stress conditions (**Figure 3**).

Focusing solely on the Solanaceae plant family, melatonin and ABA are vital for enhancing tomato salt stress tolerance and exhibit interconnected functions (Hu et al., 2021). Tomato seedlings treated with melatonin, ABA, and Melatonin + ABA after exposure to 200 mM NaCl exhibited enhanced antioxidant capacity, reduced ROS, and MDA levels, elevated proline, endogenous melatonin, photosynthetic pigments, and up-regulated redox, salt tolerance-related genes, and melatonin biosynthesis genes. ABA catabolism increased, and anabolism decreased, lowering endogenous ABA. The treatments differed in mechanisms; melatonin improved the AsA-GSH cycle and stomatal conductance, ABA increased proline, and reduced stomatal conductance, while melatonin + ABA regulated hormone metabolism and improved salt-regulated gene expression. Another study investigated how external melatonin protects against drought-induced carbonyl/oxidative stress and enhances antioxidative defense systems in potato plants (El-Yazied et al., 2022). It influences by inhibiting ABA transport from roots to shoots to regulate tuberization and simultaneously increasing non-reducing sugar essential for sugar transport and starch synthesis in tubers.

Last but not least important, (Jahan et al., 2021b) highlighted that heat stress and ABA induce leaf senescence, but melatonin and GA are vital in suppressing this process. However, the specific mechanism underpinning the signaling relationship between MT, GA, and ABA in heat-induced leaf senescence remains largely unexplored. This study aimed to investigate melatonin’s potential in preventing heat-induced leaf senescence in tomatoes, and how ABA and GA interact in its presence. Tomato seedlings were pretreated with 100 µM melatonin or water and then exposed to high temperatures (38/28°C) for 5 days. Heat stress hastened senescence by damaging photosystems and increasing ROS, particularly *RBOH* gene expression. Conversely, melatonin treatment significantly mitigated heat-induced leaf senescence, evident in reduced leaf yellowing, an enhanced Fv/Fm ratio (indicating photosystem efficiency), and lower ROS production. Furthermore, melatonin treatment notably restrained heat-induced leaf senescence, suppressing the *Rbohs* gene, chlorophyll catabolic genes, and senescence-associated gene expression levels. Importantly, ABA enhances the gene’s expression, whereas melatonin subdues it. These findings highlight the distinct roles of melatonin and ABA in tomato plants under drought, salt, and heat stress.

In conclusion, the cited studies provide ample evidence of the profound interactions between melatonin and ABA in plant stress responses. Notably, examples from both Solanaceae and non-Solanaceae plants highlight that the antagonistic response of plants to ABA and melatonin extends beyond just a single plant family. These pioneering studies underscore the potential of leveraging the melatonin-ABA interplay in horticultural practices to bolster plant resilience in the face of various abiotic stressors.

### 5.2 Experimental approaches used to assess their interplay

These studies utilized various experimental designs to dissect the complex interplay between ABA and melatonin. Notably, the exogenous application of melatonin under different stress conditions allowed for the observation of plant responses with ABA. The manipulation of melatonin and ABA levels, either through treatment or genetic alteration, provided insights into their cooperative or antagonistic roles. Measurement of specific physiological and biochemical markers, alongside growth and development observations, constituted integral parts of the studies. Furthermore, advanced molecular techniques like gene expression analysis were harnessed to shed light on the underlying molecular mechanisms linking melatonin and ABA pathways.

## 6 Molecular mechanisms and signaling pathways

The intricacies of melatonin and ABA interaction are substantially governed by the activity of a suite of significant genes. Such genes can be identified through tools like RNA-sequencing and gene knockout or overexpression studies, revealing critical components of the signaling pathways and allowing for further characterization (Shukla et al., 2021). A gene like *SNRK2*, for example, is crucial in the ABA signaling pathway and was found to also interact with melatonin, signifying its role in mediating the crosstalk (Mansouri et al., 2021). Moreover, the transcriptional regulation, marked by shifts in gene expression patterns, provides further insights into the molecular interplay between melatonin and ABA. For instance, studies have shown the upregulation of stress-responsive genes like *RD29A* and *RD29B* under abiotic stress, following exogenous melatonin application. Meanwhile, ABA-responsive elements (ABREs) in the promoters of these genes play a critical role, indicating the convergence of these two signaling pathways (Shukla et al., 2021).

The response of various plant species to the interplay between melatonin and ABA can be better understood through the use of gene expression patterns (Shi et al., 2015). Methods that rely on gene expression patterns, however, depend heavily on protein-to-protein interactions, and post-translational modifications (or the lack thereof). Examples of these would be the interactions of protein kinases, phosphatases, regulatory proteins, and transcription factors. For instance, the modulation of *SNRK2* activity via phosphorylation, a post-translational modification, has been implicated in both melatonin and ABA signaling, influencing plant stress responses (Mansouri et al., 2021).

The knowledge derived from these aspects provides profound insights into the regulatory network that controls plant stress responses. The cooperation and sometimes competition between melatonin and ABA in response to different stressors is largely a product of the interplay between multiple levels of biological regulation - from the genetic to post-translational. Understanding the molecular mechanisms, their interconnections, and influence on plant physiology, allows us to envisage a network of processes that effectively control plant responses to various abiotic stressors (Debnath et al., 2018; Altaf et al., 2023b).

The complexity of melatonin-ABA interaction under stress conditions can be traced back to their common and divergent roles in the regulation of key genes, transcriptional control, protein interactions, and modifications (Yang et al., 2015). By further investigating these areas, we may elucidate the intricate signaling networks underpinning abiotic stress responses in Solanaceae crops, providing opportunities for stress resilience improvement.

## 7 Applications and implications for horticultural practices

The intricate interplay between melatonin and ABA interaction has far-reaching implications for designing novel strategies to bolster stress tolerance in crops. An in-depth understanding of these molecular interactions could inspire the development of genetically engineered crops capable of effectively utilizing these two hormonal pathways. Biotechnological advances could facilitate the genetic manipulation of key genes involved in the crosstalk, enhancing crop stress tolerance without compromising growth or yield.

Moreover, integrating knowledge of the melatonin-ABA interaction into conventional breeding programs could also be advantageous. Breeders could select for traits associated with the improved efficiency of these two signaling pathways, such as faster recovery from stress or reduced damage under stressful conditions (Fu et al., 2017; Tan et al., 2019; Hu et al., 2021). Considering the polygenic nature of stress tolerance traits, marker-assisted selection and genomic selection techniques could be utilized to improve the efficiency of breeding programs. By harnessing the potential of these hormonal interactions, we could breed crops that not only withstand harsh conditions but also thrive in them.

Apart from genetic and breeding approaches, the direct application of melatonin and ABA could also prove beneficial in horticultural practices. Their exogenous application could augment the plant’s innate stress responses and provide an immediate solution to abiotic stress problems (Arnao and Hernández-Ruiz, 2021). For instance, previous studies have shown that exogenous application of melatonin can enhance ABA-induced stomatal closure, leading to improved water use efficiency under drought stress (Li et al., 2015; McAdam et al., 2017). Such practical interventions can be customized based on the type of crop and specific environmental conditions, offering a flexible and versatile solution to improve plant stress tolerance.

Nevertheless, while these strategies hold great promise, we must consider potential trade-offs and account for whole-plant and agroecosystem dynamics. We need a more profound understanding of how these hormones interact under different environmental conditions and in different plant species to optimize these strategies.

The intricate interaction between melatonin and ABA in response to abiotic stress offers promising avenues for the development of stress-tolerant crops. By combining biotechnological, breeding, and practical horticultural approaches, we could harness the potential of these hormonal interactions to develop crops that can thrive in an increasingly challenging agricultural environment. Thus, further research into the melatonin-ABA crosstalk could profoundly impact sustainable agriculture and global food security.

## 8 Future perspectives and research directions

Despite the significant strides made in understanding the roles and interplay of melatonin and ABA in Solanaceae plants’ stress responses, we have only scratched the surface of this complex biochemical interaction. Several knowledge gaps persist, opening avenues for further exploration.

A more comprehensive understanding of the molecular mechanisms underlying the crosstalk is required, specifically how the two hormones orchestrate their effects at the transcriptional, translational, and post-translational levels. Are there any undiscovered components of the signaling pathways? Do any other plant hormones contribute to this regulatory network? These are questions yet to be answered.

Moreover, the use of mutant or transgenic plants could be another effective approach to investigate the functionality and contribution of specific genes in the crosstalk. By comparing these plants with their wild-type counterparts, the roles of individual genes can be better understood.

Crucially, it is essential to continue studying Solanaceae plants in this context. Solanaceae, or nightshade plants, are not only agriculturally important, but they also exhibit high melatonin levels. Hence, they provide a unique opportunity to study melatonin’s role in stress responses and its interplay with ABA. The findings from such studies could potentially be extended to other plant families, thus having broader agricultural implications.

Therefore, the complexity of the melatonin-ABA interaction requires a comprehensive, multifaceted research approach. Through continued investigation into this crosstalk in Solanaceae plants and the application of advanced research methodologies, we can fill in the existing knowledge gaps. By doing so, we will be one step closer to harnessing this interaction for crop improvement and sustainable agriculture, ultimately contributing to global food security.

## 9 Conclusions

In conclusion, unraveling the implications of the melatonin-ABA interplay in abiotic stress responses holds profound significance for Solanaceae plants. From genetic and breeding strategies to innovative agronomic practices and broader implications for sustainable agriculture, this knowledge holds promise for enhancing stress tolerance and ensuring food security in a changing climate. The significance of melatonin and ABA interactions is evident across diverse plant functions, including seed germination, seedling growth, stomatal behaviors, antioxidant defense, fruit ripening, and improvement of abiotic stress resistance. A deeper understanding of the molecular mechanisms underlying the interplay between melatonin and ABA in abiotic stress management among the Solanaceae plant family would be valuable for identifying and manipulating key genes involved in managing problematic stress responses. Moreover, these findings can facilitate advancements in agronomic practices, guiding better protection strategies for Solanaceae plant species against abiotic stressors. This is achieved by leveraging insights into their responses to melatonin and ABA interactions, encompassing both cooperative and antagonistic dynamics. Enhancing our comprehension of this intricate hormonal interplay can unlock the potential for improved crop performance, contributing to a more resilient and sustainable agricultural future. However, the melatonin and ABA interplay possess substantial untapped potential, and several of its potential advantages for the horticultural industry remain not fully understood. Therefore, exploring potential applications of melatonin in agricultural practices, to improve crop yield under challenging environmental conditions, warrant further investigation. These findings provide essential insights into the plausible applications of melatonin in agricultural practices, to enhance crop output under challenging environmental conditions. Understanding the complex relationships between melatonin and other plant hormones can lead to innovative approaches for bolstering stress tolerance and optimizing plant growth and development in the face of environmental stressors. Further research in this area could result in novel methods for achieving sustainable agriculture and ensuring food security.

## Author contributions

MA: Conceptualization, Data curation, Formal Analysis, Investigation, Methodology, Software, Writing – original draft, Writing – review & editing. YP: Conceptualization, Validation, Visualization, Writing – review & editing. HL: Conceptualization, Investigation, Validation, Visualization, Writing – review & editing. ZC: Conceptualization, Funding acquisition, Project administration, Resources, Supervision, Validation, Writing – review & editing.
